# Physicians, Primary Caregivers and Topical Repellent: All Under-Utilised Resources in Stopping Dengue Virus Transmission in Affected Households

**DOI:** 10.1371/journal.pntd.0004667

**Published:** 2016-05-10

**Authors:** Nguyet Minh Nguyen, James S. Whitehorn, Tai Luong Thi Hue, Truong Nguyen Thanh, Thong Mai Xuan, Huy Vo Xuan, Huong Nguyen Thi Cam, Lan Nguyen Thi Hong, Hoa L. Nguyen, Tam Dong Thi Hoai, Chau Nguyen Van Vinh, Marcel Wolbers, Bridget Wills, Cameron P. Simmons, Lauren B. Carrington

**Affiliations:** 1 Oxford University Clinical Research Unit, Ho Chi Minh City, Vietnam; 2 Hospital for Tropical Diseases, Ho Chi Minh City, Vietnam; 3 University of Medicine and Pharmacy of Ho Chi Minh City, Ho Chi Minh City, Vietnam; 4 Centre for Tropical Medicine and Global Health, Nuffield Department of Clinical Medicine, University of Oxford, Oxford, United Kingdom; 5 Department of Microbiology and Immunology, University of Melbourne, The Peter Doherty Institute for Infection and Immunity, Melbourne, Australia; Common Heritage Foundation, NIGERIA

## Abstract

**Background:**

Primary health care facilities frequently manage dengue cases on an ambulatory basis for the duration of the patient’s illness. There is a great opportunity for specific messaging, aimed to reduce dengue virus (DENV) transmission in and around the home, to be directly targeted toward this high-risk ambulatory patient group, as part of an integrated approach to dengue management. The extent however, to which physicians understand, and can themselves effectively communicate strategies to stop focal DENV transmission around an ambulatory dengue case is unknown; the matter of patient comprehension and recollection then ensues. In addition, the effectiveness of *N*,*N*-diethyl-3-methylbenzamide (DEET)-based insect repellent in protecting dengue patients from *Aedes aegypti* mosquitoes’ bites has not been investigated.

**Methodology:**

A knowledge, attitude and practice (KAP) survey, focusing on the mechanisms of DENV transmission and prevention, was performed using semi-structured questionnaires. This survey was targeted towards the patients and family members providing supportive care, and physicians routinely involved in dengue patient management in Southern Vietnam. An additional clinical observational study was conducted to measure the efficacy of a widely-used 13% DEET-based insect repellent to repel *Ae*. *aegypti* mosquitoes from the forearms of dengue cases and matched healthy controls.

**Principal Findings:**

Among both the physician (*n* = 50) and patient (*n* = 49) groups there were several respondents lacking a coherent understanding of DENV transmission, leading to some inappropriate attitudes and inadequate acute preventive practices in the household. The application of insect repellent to protect patients and their relatives from mosquito bites was frequently recommended by majority of physicians (78%) participating in the survey. Nevertheless, our tested topical application of 13% DEET conferred only ~1hr median protection time from *Ae*. *aegypti* landing. This is notably shorter than that advertised on the manufacturer’s label. No differences in landing time between febrile dengue cases or matched healthy controls (*n* = 19 experiments) were observed.

**Conclusion/Significance:**

Our study identifies missed opportunities for primary care physicians to improve public health through communication of strategies that could prevent focal dengue transmission in and around a case household. We advocate better access to more efficient communication methods for physicians and auxilliary health workers, supporting to educate those at high risk of DENV transmission. Our empirical testing of a widely-available 13% DEET-based repellent was limited in its protective efficacy against *Ae*. *aegypti* mosquito bites, and therefore DENV transmission, suggesting more frequent application is necessary to be beneficial.

## Author Summary

In endemic countries many dengue cases are managed on an ambulatory basis throughout their illness. Many of these ambulatory cases will be infectious to *Ae*. *aegypti* mosquitoes for some period of the febrile phase. A survey of two key actors, physicians who manage dengue cases and the primary caregiver to dengue cases, was performed to assess their knowledge, attitudes and practices on the mechanism of DENV transmission and prevention. In addition, the duration of protection from mosquito landing was measured and compared between febrile dengue patients and healthy volunteers using a locally available, and widely-used 13% *N*,*N*-Diethyl-3-methylbenzamide (DEET)-based topical insect repellent. This KAP survey demonstrated an incomplete understanding of DENV transmission among several patients and physicians, leading to some inappropriate attitudes and inadequate preventive practices in the household. This suggests that aspects of the knowledge, communication between physicians *and* patients about dengue, and what patients have retained from the clinical consultation, can be improved. The use of topical insect repellent to protect patients and relatives from mosquito bites was recommended by a large proportion of physicians. However, the repellent used in this experiment provided only a modest repellent effect to *Ae*. *aegypti* mosquitoes, with no difference in protection time when applied to either dengue patients or healthy controls. Future recommendations to patients should continue to promote repellent use, but advocate much more frequent application. To directly target those patients at risk of onward DENV transmission, this research highlights the potential benefits of physicians imparting practicable and effective advice on preventive measures of dengue case management, as part of a holistic approach to dengue control. Finally, findings of this work can provide data for approaching effectively preventive practices for DENV and other flaviviruses transmitted by *Aedes* spp.

## Introduction

Dengue is the most important arboviral infection of humans globally and is primarily transmitted between humans through the bites of *Aedes aegypti* mosquitoes [1,2]. It is estimated that there are approximately 390 million infections annually, and 3.9 billion people living at risk of dengue virus (DENV) infection [3]. Vietnam is a country with a 50+ year history of sustained DENV transmission. Dengue epidemics in this country were first reported in 1959 in northern Vietnam [4]. Since then, Vietnam has become a country with hyperendemic DENV transmission (i.e.: with the co-circulation of all four serotypes of DENV), and an increasing annual incidence of hospitalized dengue cases throughout the country [5–7]. Dengue prevention has now entered a new era with a live, attenuated, tetravalent dengue vaccine of Sanofi-Pasteur recently licensed in Mexico, Brazil and the Philippines. However, in Vietnam and many other countries where dengue is endemic, the vaccine has not yet been licensed, and mosquito control therefore remains the mainstay for preventing DENV transmission in the community.

Symptomatic dengue patients had been found to transmit DENV to *Aedes* spp. mosquitoes from ~1.5–2 days prior to fever onset until day 3–6 of illness [8–10]. Recently, Nguyet et al. [11] and Whitehorn et al. [12] have identified the plasma DENV viremia levels in Vietnamese dengue patients needed to successfully infect 50% of exposed *Ae*. *aegypti* or *Ae*. *albopictus* mosquitoes (50% mosquito infectious dose; MID_50_). In addition, most ambulatory dengue cases were revealed to have viremia levels that exceed the MID_50_ in the first few days of their illness [11], indicating that most are still infectious to *Ae*. *aegypti* when they return to their homes. Epidemiological studies also confirm the home is a location of risk with respect to DENV transmission [13,14]. The capacity of asymptomatically-infected individuals to transmit to *Ae*. *aegypti* has been recently highlighted, but more work is needed to understand their prevalence and importance to the overall epidemiology of dengue [10].

Physicians managing dengue cases in outpatient or primary care settings, are ideally placed to communicate how best to reduce the risk of DENV in an affected household. Previous studies have explored the Knowledge, Attitude and Practice (KAP) of communities affected by dengue, but surprisingly, such studies have never specifically targeted physicians who manage cases, nor the affected dengue patients or their primary caregivers [15–22]. A robust understanding of how DENV is transmitted between humans, and what interventions can effectively reduce the risk of further dengue transmission is central to the success of physician-led initiatives. An equally important issue is the extent to which patients and their families are able to understand and act upon the information given by their primary care physicians.

Mosquito repellents containing N,N-Diethyl-3-Methylbenzamide (DEET) are the most effective insect repellent [23–29]. Dengue patients, like other febrile patients, may be more attractive to mosquitoes compared to non-febrile individuals due to their higher body temperature, and therefore higher production of carbon dioxide and possibly other volatile compounds [30–32]. As a result, the mosquito repellents may afford reduced protection for febrile dengue patients compared to healthy individuals.

The aims of this study were a) to describe the general KAP of physicians and patients regarding dengue and DENV transmission; b) to understand the physicians perception of what they communicate to patients regarding local prevention of dengue transmission; and c) to investigate whether patients understand and recall the information provided by their physicians and then act upon these suggestions. Finally, we aimed d) to determine the efficacy of a DEET-containing topical mosquito repellent widely-used in Vietnam, and assess whether efficacy differed between febrile dengue patients and non-febrile healthy controls. Findings from this study can improve dialogue about dengue and DENV transmission between physicians and their patients, by highlighting barriers in communication and knowledge gaps.

## Materials and Methods

### Ethical considerations

Protocol and questionnaires of the KAP survey were reviewed and approved by the Ethics Committee of Hospital for Tropical Diseases (HTD), Ho Chi Minh City, Vietnam (CS/ND/14/25). This survey was considered low risk, hence did not need formal approval from the Oxford University Tropical Research Ethics Committee (OxTREC). The topical repellent study was approved by the Ethics Committee of HTD (CS/ND/12/12) and the OxTREC (OxTREC 66–11). All patients and healthy volunteers gave written informed consent to participate in the repellent study.

### KAP survey

#### Participant enrolment for the KAP survey

The KAP survey was targeted toward Vietnamese physicians and dengue patients or their primary caregivers and was focused on their understanding of DENV transmission and prevention. The first group of participants were physicians working in primary care practices or tertiary referral hospitals in Ho Chi Minh City; or at provincial hospitals in southern Vietnam. All physicians provided consent before participating in the survey, which was conducted between September and November 2014 either electronically online (https://www.surveymonkey.com), or using a paper-based questionnaire. The second group consisted of clinically suspected dengue patients or primary caregivers of those under 15 years old. The patients were identified through their enrolment, between September and December 2014, into one of two ongoing descriptive studies taking place at the outpatient departments of Children’s Hospital Number 1 (CH#1), Children’s Hospital Number 2 (CH#2) or HTD in Ho Chi Minh City, Vietnam. Study staff, blinded to the laboratory diagnostic test results of patients, contacted eligible patients or caregivers, either via phone calls or in person when they came back for follow-up between 10 and 14 days after enrolment into one of these studies, and invited them to participate in the survey. Participants in the patient group provided verbal consent before responding to the questionnaire. Recruitment for this group was based on the list of patients who came back for follow-up visit, and the availability of human resources. The responses of all participants (physicians and patients) in this survey were self-reported.

#### Questionnaires

The semi-structured questionnaires were based on previous KAP surveys, but were modified to more specifically understand the communication between physicians and patients about DENV transmission and prevention [17,21,22]. Questionnaires were originally developed in English. They were then translated into Vietnamese by a bilingual staff member, and back-translated into English by a professional translator for verification. We pre-tested the questionnaire on a small number of volunteers to receive feedback on content before they were finalized.

### Topical mosquito repellent study

This observational study assessed the effectiveness of a commercial 13% *N*,*N*-Diethyl-3-methylbenzamide (DEET)-based repellent (“Soffell”, Fountain of Youth, Pty Ltd, Singapore) against the landing of *Ae*. *aegypti* mosquitoes, on Vietnamese adult febrile dengue patients compared to adult healthy controls, using an arm-in-cage method [26].

#### Participant enrolment for the repellent study

Patients admitted to one of two specialist dengue wards at HTD between October 2013 and December 2014, were eligible for enrolment if they fulfilled the inclusion and exclusion criteria. Inclusion criteria were a) adult patients (≥ 18 years of age) admitted within the first 96 hours of fever with clinical suspicion of dengue; b) a positive NS1 rapid test; and c) written informed consent. The exclusion criteria were a) pregnancy (either clinically confirmed, or by urine dipstick for human chorionic gonadotropin hormone); b) patients in intensive care unit or those considered clinically instable by their attending physician; c) learning disability (or any other condition that might risk the patient’s ability to give informed and autonomous consent); and d) history of severe dermatological conditions or severe reactions to mosquito bites.

Dengue patients were matched to healthy controls for age (+/- 5 years), sex and body mass index (BMI) (+/- 2). The inclusion criteria for healthy controls were a) adults (≥18 years of age); and b) written informed consent. Exclusion criteria were a) pregnancy; b) current febrile illness; and c) history of severe dermatological conditions or severe reactions to mosquito bites.

#### Mosquito exposure

**Fig 1:**
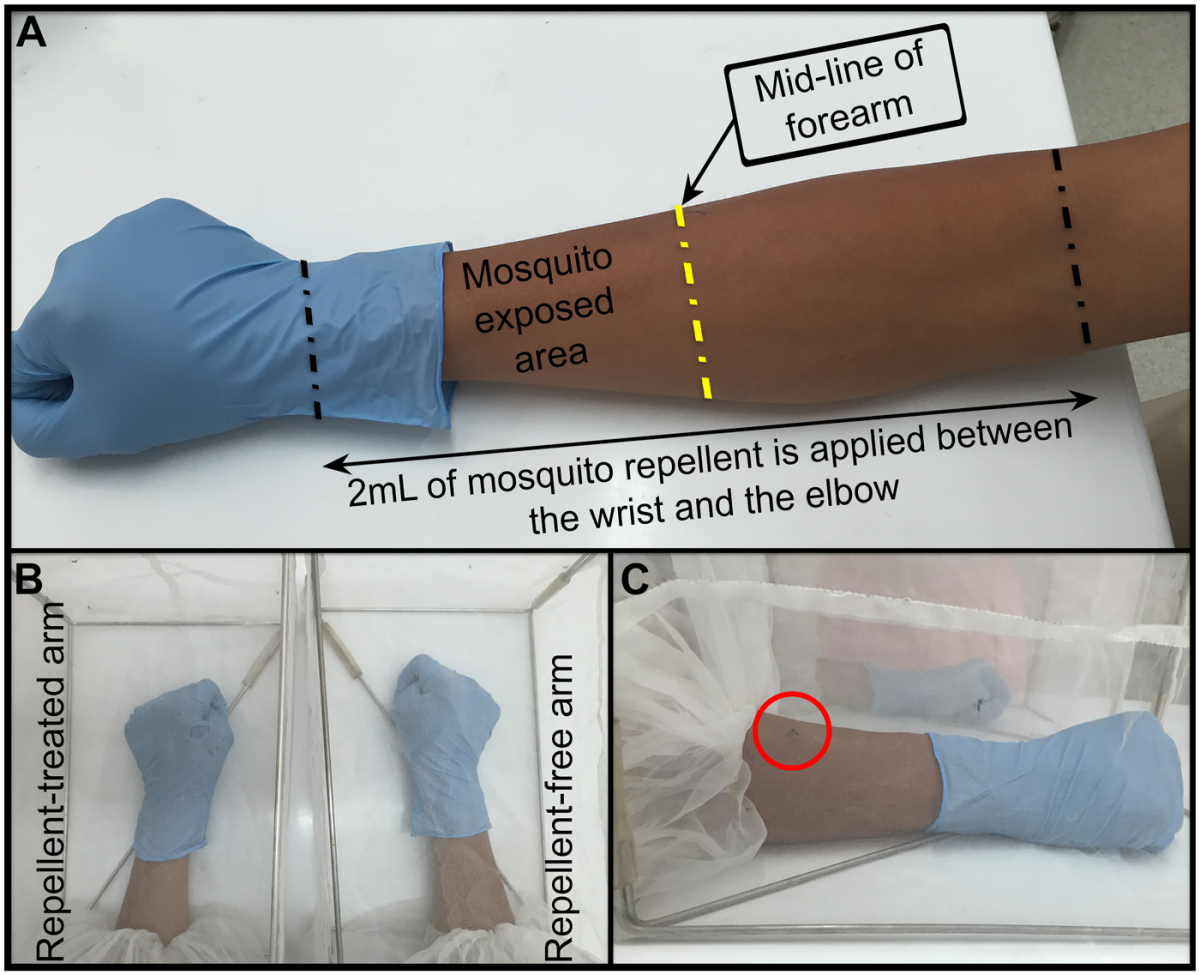
Annotated photos of the “arm-in-cage” method. **(A)** Mosquito repellent (2 mL) was applied onto all sides of a single forearm, between the elbow and wrist (repellent-treated arm). The opposite limb had no repellent applied, and was used as an internal control (repellent free arm). **(B)** The participant inserted her/his arms into the two mosquito cages simultaneously. Each cage contained ten *Aedes aegypti* mosquitoes. **(C)** A mosquito (circled) landing on a participant’s arm during an exposure. The arms were exposed for a maximum of 2 minutes each time. “Landing for 2 seconds” was used as a proxy for mosquito biting. After a mosquito landed on a participant, they were asked to shake off the mosquito and withdraw their arm from the cage. Alternatively, the participant withdrew their arms only at the end of the two-minute exposure if there was no mosquito landing, and the exposure was repeated at the next scheduled interval.

The effectiveness of topical repellent was evaluated using a previously described, arm-in-cage method (Fig 1) [26]. Repeated brief mosquito exposures (for up to 2 minutes) were performed following the schedule shown in S1 Fig. The *Ae*. *aegypti* mosquitoes used were field-derived, F_3_, 4–8 day old females that had not yet been either blood fed or exposed to any form of insect repellent. The previous parental generation of mosquitoes were confirmed free of DENV, chikungunya virus, and Japanese encephalitis virus. All mosquitoes were starved for 16–24 hours prior to the commencement of experiments. Four cages (30 cm x 20 cm x 20 cm), each containing ten *Ae*. *aegypti* mosquitoes, were randomly assigned to each participant (dengue case and matched control). One cage was used for the repellent-free arm (with the expectation that a mosquito landing would occur within the first two-minute exposure). The other three cages were used for the repellent-treated arm. The randomised use of these three cages was to reduce any bias associated with using only one cage and to prevent accumulation of repellent on or around the single cage, which may prolong the real duration of effectiveness of repellent. Study staff applied 2 mL of repellent onto all sides of a single forearm, between the elbow and ulnar prominence, for each participant. The forearm on which the repellent was applied (left or right) was randomised, and could differ between the two participants in each experiment. No repellent was applied to the opposite limb, which was used as an internal control for each participant. Tympanic temperature was measured both before the exposure and at the time of mosquito landing on the repellent-treated arm, for both participants. Mosquitoes used in each experiment were all killed when the experiments were finished, and there was no transfer of mosquito cages between participants.

During each 2-minute exposure we used “landing for 2 seconds” as a proxy indicator of mosquito biting, and therefore landing was taken as “breakthrough” of the repellent effect. Two observers watched to see if a mosquito landed on participants’ arms. Upon a 2-second landing, the observers asked participants to shake off the mosquitoes from that arm, and remove it from the cage. The experiment continued with the other arm until a mosquito landed on it. Observers used a stopwatch for recording the time taken for a mosquito to land on each arm, measured to the nearest second.

### Data analysis

Baseline characteristics of participants were described with categorical variables presented as proportion, and continuous variables reported as median (with interquartile range [IQR]). Responses of physicians and patients for each question were presented as the proportion of respondents who selected each answer.

The distribution of the time to mosquito-landing in febrile dengue cases and healthy controls, respectively, was estimated using the Kaplan-Meier method. The effectiveness of repellent for each participant was calculated as the difference in time elapsed at landing of mosquitoes between the repellent-treated and non-treated arms. The time to landing in the repellent and the non-repellent arms, respectively, were compared between dengue patients and controls with a Cox Proportional Hazard regression model. Robust standard errors were used to account for the matched design. A similar Cox model was used to assess the influence of body temperature on the time to landing.

All data analyses were performed with the statistical software R, version 3.1.1 (R Foundation for Statistical Computing, Vienna, Austria).

## Results

### KAP survey of physicians

**Fig 2:**
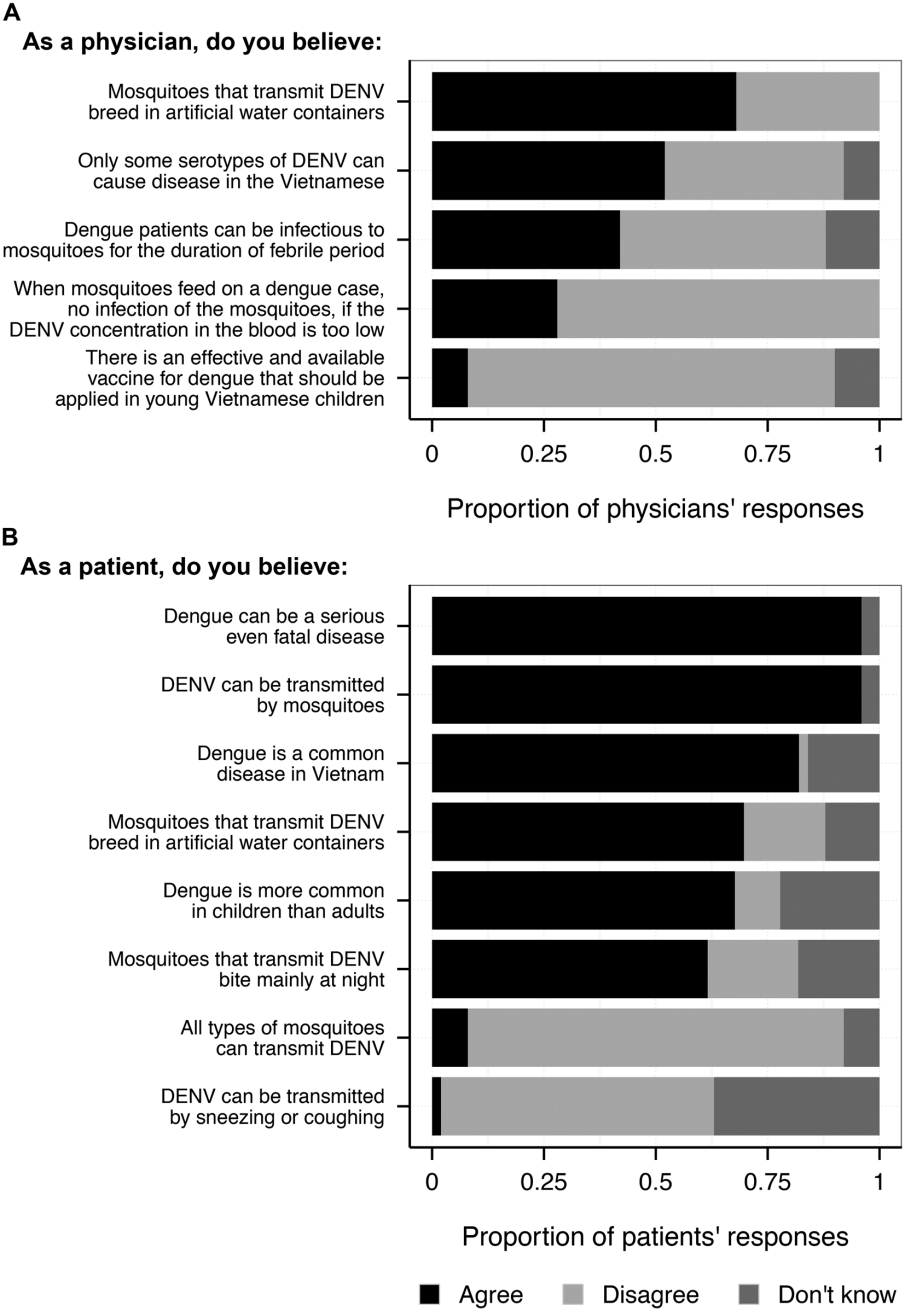
Survey responses of (A) physicians and (B) patients on general knowledge of DENV tranmission and prevention. Black bars represent the proportion of responses given by participants, who agreed with the statement. The light-grey part of each bar represents the proportion of participants, who did not agreed with the statement. The dark grey bars show the proportion of participants who reported that they did not know the response to that question.

A total of 70 physicians were invited to join the survey; 16 declined to participate and four failed to complete the questionnaire, resulting in 50 questionnaires available for analysis. The profile of the responding physicians, including their clinical specialties, is shown in Table 1. Most physicians said that the primary DENV vector mainly breeds in artificial water containers (68%), and that an effective vaccine for dengue is not currently available (82%). Interestingly, seven (14%) physicians thought that *Anopheles* spp. mosquitoes are the primary vector of DENV (S1 Information). In addition, 52% of physicians believed that only some serotypes of DENV could cause disease. Regarding the likelihood of mosquitoes becoming infected with DENV when blood-feeding on dengue patients, 29 physicians (58%) thought that the mosquitoes would immediately become infected with DENV and could transmit the virus to other people in their next bites, while 14 (28%) thought that the mosquitoes could not be infected if the concentration of DENV in the patient’s blood was too low. There were 21 (42%) respondents who believed that dengue patients could be infectious to mosquitoes throughout the febrile phase of their illness, whereas 4 (8%) thought that infectiousness lasted for 1–2 days; 19 (38%) thought it could last for 1–2 weeks and the remaining six respondents (12%) said that they did not know for how long dengue patients could be infectious to mosquitoes (Fig 2 and S1 Information).

**Table 1:** Demographic characteristics of physicians who responded to the KAP survey.

Characteristics	*n* = 50
Age, years—median (IQR)	43 (33–51)
Male sex, *n* (%)	28 (56)
Specialty, *n* (%)	
Infectious diseases	12 (24)
Pediatrics	13 (26)
Internal medicine	19 (38)
Others (emergency, dermatology, neurology, neurosurgery)	6 (12)
Frequency of working at outpatient department, *n* (%)	
Every day	11 (22)
One day per week	8 (16)
Two days per week	9 (18)
Three days per week	3 (6)
Four days per week	4 (8)
Others (less than once a week)	8 (16)

**Fig 3:**
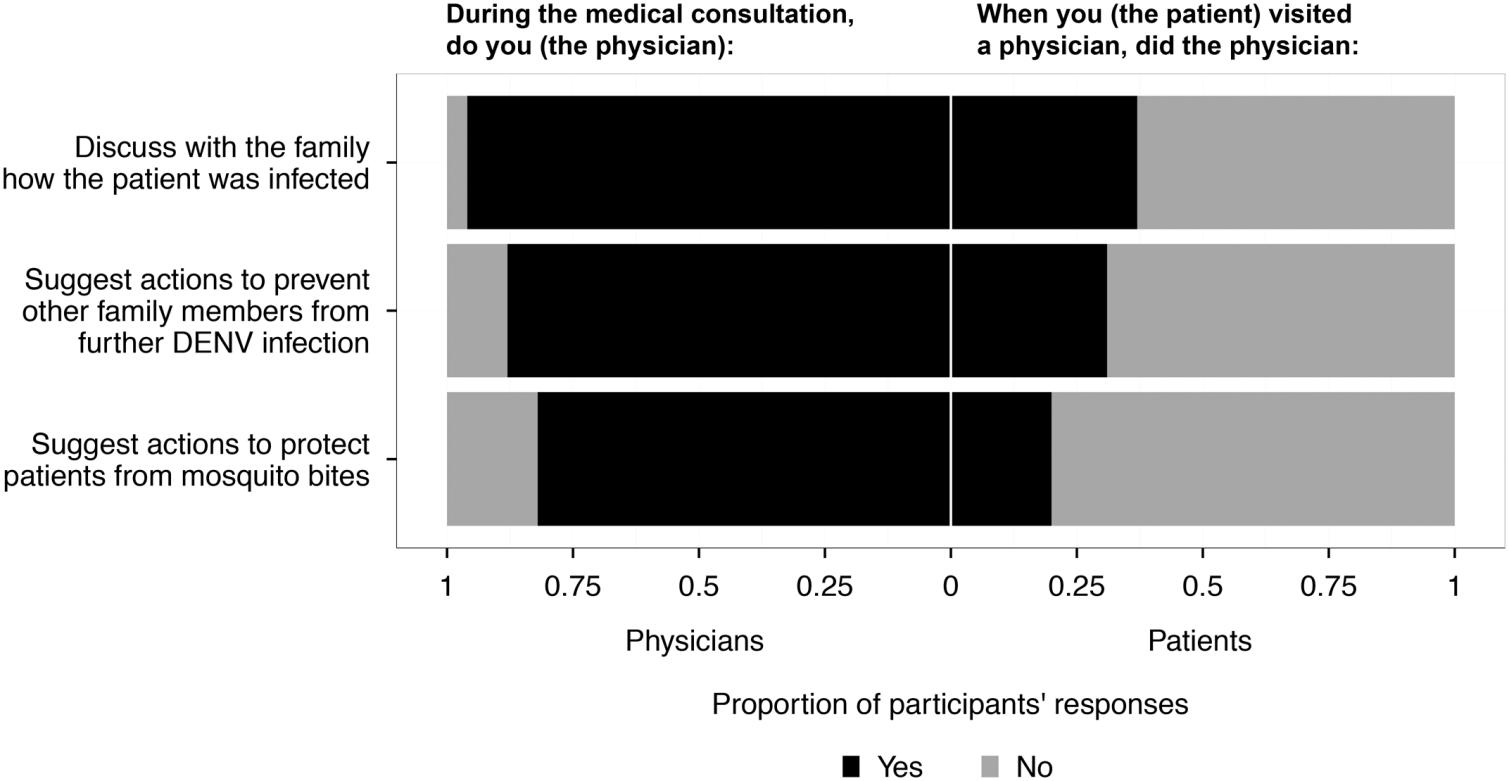
Dissemination of information by physicians and recollection of information by patients. The left side of the graph shows the responses of the physicians when asked if they discuss with their patients certain topics. The right hand side of the graph illustrates whether the patients understood or recall this same information being given to them during their consultation. (NB: the physicians and patients surveyed are independent; they did not have consultations with each other). Black and light-grey bars represent the positive and negative responses respectively. A list of recommendations suggested by physicians can be found in S1 and S2 Information.

Almost all respondents stated that dengue was a very important disease in their daily clinical practice (92%), and believed it was their responsibility to discuss DENV transmission and prevention with patients and caregivers (94%). The majority reported that they routinely provide specific information regarding how the patient was infected (96%) and how to prevent patients from receiving further bites from *Aedes* spp. (82%), as well as how to reduce the risk of DENV infection for other people living in the same house (88%) (Fig 3). An integrated programme of interventions comprising use of mosquito bed nets (100%), personal mosquito repellents (78%), and insecticide spraying of adult mosquitoes (78%) was commonly recommended by physicians to protect dengue patients from subsequent *Aedes* spp. bites. In terms of reducing risk of DENV infection in the household, removing mosquito-breeding sites was the most common recommendation (98%), followed by avoiding mosquito bites (86%) and killing adult mosquitoes (82%).

### KAP survey of patients and their caregivers

Patients (*n* = 4) and the primary caregivers of those who were under 15 years of age (*n* = 47 independent cases) were approached for interview at a median of 14 (IQR = 12–14) days after their initial enrolment into one of the two outpatient studies. Survey responses from two participants (both of whom were caregivers) were excluded because they did not complete the survey. Therefore, in total 49 questionnaires were used for further analysis. Baseline characteristics of respondents in this group are presented in Table 2. Among these 49 patients, 22 (45%) had laboratory confirmation of a DENV infection. The majority of respondents agreed that dengue is a common disease in Vietnam (82%) and that this disease could be serious, even life-threatening (96%).

**Table 2:** Demographic characteristics of participants in the patient group who responded to the KAP survey.

Characteristics	*n* = 49
Age of respondents, years—median (IQR)	37 (33–41)
Male sex of respondents, *n* (%)	14 (28)
Respondents group, *n* (%):	
Patients	4 (8)
Caregivers (parents or guardians)	45 (92)
Occupation, *n* (%):	
Vendor	20 (41)
Housework man/woman	12 (24)
Factory worker	12 (24)
Office staff	2 (4)
Teacher	1 (2)
Farmer	1 (2)
Others—security guard	1 (2)
Educational level, *n* (%):	
Elementary school	7 (14)
High school	35 (72)
Higher	7 (14)

Ninety-six percent of respondents (*n* = 47) believed that DENV could be transmitted between humans through mosquito bites, among those 4% (*n* = 2) believed that DENV are transmitted by all types of mosquitoes while 9% (*n* = 4) said that they did not know about this statement and 87% (*n* = 41) disagreed with the statement. In regards to knowledge on the breeding sites of the mosquito vectors, 72% (*n* = 34) stated they bred primarily in artificial water containers (Fig 2). A further 60% (*n* = 28) said the mosquitoes mainly bite humans at night; only 21% disagreed with this statement (S2 Information), and 77% (*n* = 36) reported routinely performing control measures to reduce mosquito abundance within their home (at least once a week). Killing mosquitoes and removing larval breeding sites were routinely performed by 47.2% of households (S2 Information).

Among *all* 49 respondents 61% (*n* = 30) believed that DENV-infected people were not contagious to others through the respiratory tract, i.e. through coughing or sneezing; 2% (*n* = 1) believed that this could be another potential mode of DENV transmission in the community, while the remaining 37% (*n* = 18) of participants said that they did not know.

### Understanding the physician-patient interaction

**Fig 4:**
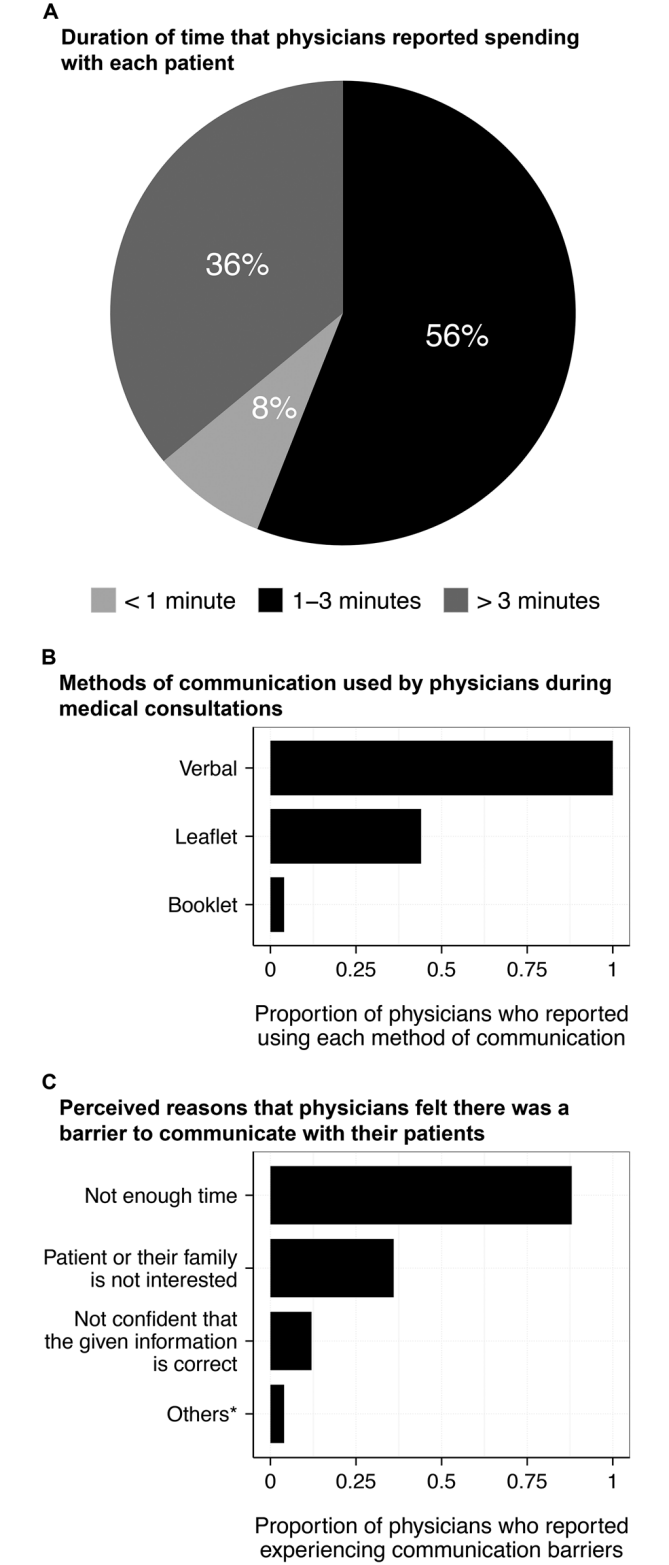
Duration, methods and perceived barriers of communication between physicians and patients during consultation. **(A)** The amount of time physicians report to spend with each of their patients to discuss their illness, during a standard medical consultation. **(B)** Modes of communication commonly used by physicians to explain DENV transmission and prevention to their patients. **(C)** Perceived barriers that physicians experience when discussing with dengue patients’ families about DENV transmission and its prevention. *Other barriers included the physicians’ feeling they lacked the training on DENV transmission cycle and prevention; and that the role of transferring information about disease prevention is forgotten during the medical consultation.

All physicians reported that they delivered information verbally about DENV transmission and prevention during their medical consultation with patients. Leaflets (44%) and booklets (4%) were also reportedly used as additional methods. Nonetheless, 56% of physicians (n = 28) said that they could spend only 1–3 minutes to perform the entire medical consultation, including discussion of the diagnosis, management and any steps to stop further transmission. Only 18 (36%) physicians were able to spend more than 3 minutes with each patient. In turn, most physicians (88%) felt that time was the most common barrier to greater discussion of DENV transmission and disease prevention. Patients’ perceived levels of interest (36%) and physicians’ self-confidence (12%) were also considered barriers of disseminating information between physicians and patients. Moreover, physicians also reported that they lacked the training on DENV transmission cycle and prevention (*n* = 1); and that the role of transferring information about disease prevention is forgotten during the medical consultation (*n* = 1) (Fig 4).

Most patients (86%) stated that they had received clear and understandable information from their physicians about the patient’s health status. Yet a much smaller proportion of respondents reported not receiving any information about how DENV could be transmitted between humans (37%), the risk of DENV infection of other members in the home (41%), how to prevent subsequent mosquito bites to the patient (20%), or how to reduce the risk of DENV infection to other family members (31%) (Fig 3).

All patients who recalled that a physician had provided recommendations about how to limit mosquito exposure for the patient reported they had acted upon these suggestions (Tables 3 and 4). In 39 respondents who did not recall receiving suggestions from their physicians, 82% (*n* = 32) performed actions to prevent the patient from mosquito bites anyway. The use of mosquito bed nets was the most common action to avoid mosquito bites, taken by 81.5% (*n* = 26) of these 32 respondents. Other measures included insecticide sprays, using an electric bat to kill adult mosquitoes and applying topical insect repellent (Table 3). One family reported that one of the actions they performed to prevent DENV transmission within their house was to isolate the dengue patient from the other family members.

**Table 3:** Actions performed in patients’ homes to prevent the *patients* from mosquito bites. The table shows the number of houses, with and without having received recommendations from the attending physician, using different methods to prevent the patient in the household being bitten by mosquitoes.

	Did your physician suggest specific actions to stop the *patients* being bitten by mosquitoes?	
	Received recommendations (*n* = 10)	Did not receive recommendations (*n* = 39)
**Number of households reported performing preventive actions, *n* (%)**	10/10 (100)	32/39 (82.0)
**Actions performed:**		
Mosquito bed net, *n* (%)	9/10 (90)	26/32 (81.5)
Electronic mosquito bat, *n* (%)	4/10 (40)	8/32 (25.0)
Insecticidal spraying, *n* (%)	8/10 (80)	16/32 (50.0)
Mosquito repellent, *n* (%)	2/10 (20)	4/32 (12.5)
Others^(^*^)^, *n* (%)	2/10 (20)	8/32 (25.0)

^(^*^)^ Other actions include: mosquito repelling coil (*n* = 3); remove breeding sites of mosquitoes (*n* = 6); remove breeding sites of mosquitoes and use coil (*n* = 1)

**Table 4:** Actions performed in patients’ homes to prevent *other family members* from DENV infection. The table shows the numbers of houses, with and without having received recommendations from the attending physician, using different methods to prevent other household members from DENV infection.

	Did your physician suggest actions that the household should take to limit the risk of DENV infection in *other family members*?	
	Received recommendations (*n* = 15)	Did not receive recommendations (*n* = 34)
**Number of households reported performing preventive actions, *n* (%)**	15/15 (100)	N/A
**Actions performed:**		
Killing adult mosquitoes, *n* (%)	9/15 (60.0)	N/A
Removing breeding sites, *n* (%)	6/15 (40.0)	
Avoiding mosquito bites, *n* (%)	13/15 (86.7)	
Others^(^*^)^, *n* (%)	1/15 (6.7)	

^(^*^)^ Other action includes the isolation of the patient from other family members (*n* = 1)

### Effectiveness of topical repellent

The effectiveness of topical insect repellent for preventing *Ae*. *aegypti* landing on either dengue cases or matched healthy controls was investigated. Of the 20 independent mosquito exposure experiments performed, 19 were assessable, and the baseline characteristics of the participants are shown in Table 5.

**Table 5:** Baseline characteristics of participants in repellent study.

Characteristics	Patients (*n* = 19)	Healthy controls (*n* = 19)
Age, years—median (IQR)	26 (20–33)	30 (28–36)
Male sex, *n* (%)	7 (37)	7 (37)
BMI, Kg/m^2^ –median (IQR)	22 (20–26)	21 (20–23)
Ethnicity, *n* (%)		
Vietnamese	19 (100)	17 (89)
Caucasian	0 (0)	2 (11)
Repellent applied to right arm, *n* (%)	10 (53)	8 (42)
Diagnosis, *n* (%)		
Dengue	16 (84)	NA
Dengue with warning signs	3 (16)	
DENV serotype, *n* (%)		
DENV-1	10 (53)	
DENV-2	0 (0)	NA
DENV-3	0 (0)	
DENV-4	9 (47)	
Tympanic temperature at mosquito release °C—median (IQR)	38.1 (37.6–38.6)	36.9 (36.6–37.2)
Tympanic temperature at landing °C—median (IQR)	38.2 (37.5–38.4)	36.9 (36.7–37.1)

**Fig 5:**
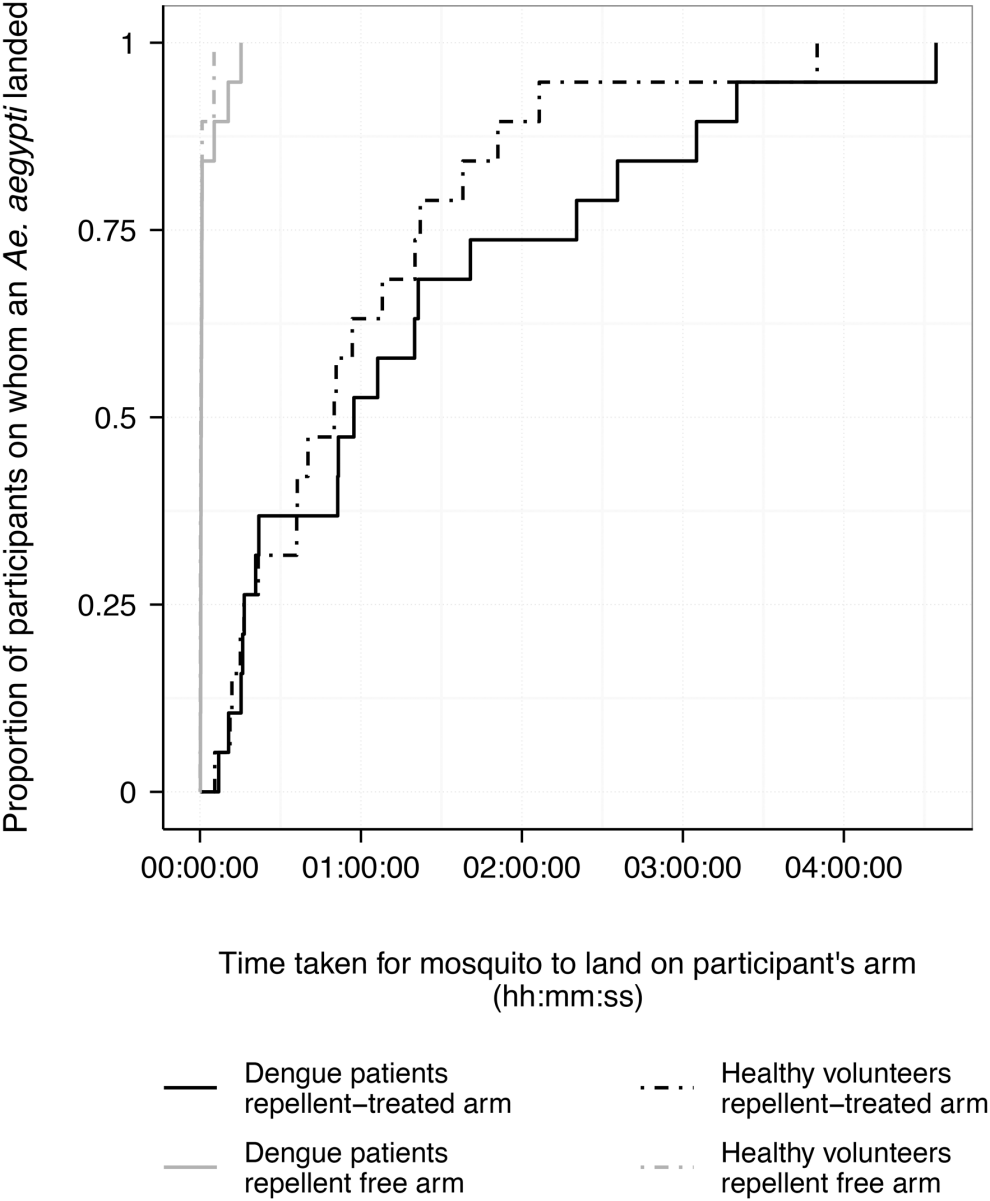
Kaplan-Meier curves showing the time-to-landing for mosquitoes on participants’ skin. Curves estimate the elapsed time to *Aedes aegypti* mosquito landing on both repellent-free and repellent-treated arms of febrile dengue patients versus non-febrile healthy controls. No statistical differences were observed between estimates for participant groups for either treatment group (repellent-treated or repellent-free arms).

The effectiveness of 13% DEET-based repellent, as measured by the median difference between landing times of repellent-treated and non-treated arms, was 49m32s (IQR = 16m01s–01h19m29s) and 56m43s (IQR = 15m55s–02h20m00s) for healthy controls and patients, respectively (Fig 5). For the 13% DEET-based repellent arms, there was no significant difference in elapsed time to landing between patients versus controls (Hazard ratio 0.71; 95% CI 0.42–1.19; *p* = 0.20), and in approximately half (11/19, 58%) of the experiments mosquitoes landed on the patient’s skin first. There was also no clear evidence that the host’s tympanic temperature affected the duration of protection from *Ae*. *aegypti* landing, irrespective of the hosts’ febrile status (Hazard ratio 0.89; 95% CI 0.61–1.29; *p* = 0.53 per +1°C increase in tympanic temperature). Further, *Ae*. *aegypti* appeared to be similarly attracted to both febrile dengue patients and healthy controls when no repellent was worn, with a median time to landing of 18s (10s–40s) in the control group versus 28s (17s–44s) in dengue patients (Hazard ratio of dengue patients vs. control: 0.64; 95% CI 0.36–1.14; *p* = 0.13).

## Discussion

In southern Vietnam, more than 60,000 dengue cases are hospitalized each year [6]. The number of clinically suspected dengue patients presenting at outpatient clinics, but whose illness does not warrant hospitalization (and therefore is not mandated for reporting to the Ministry of Health), is believed to be much higher. Some visits represent the patients’ first contact with health care services, others represent follow-up visits as part of a clinical management plan. In principle, physicians engaged in these consultations are superbly placed to deliver messages that could help stop DENV transmission within an affected household and/or to the surrounding community. Yet remarkably, this is to our knowledge, the first reported KAP survey of both physicians and patients with regard to their understanding of DENV transmission and prevention. The results demonstrate that a proportion of Vietnamese physicians involved in dengue case management lack of full understanding of DENV transmission dynamics. For instance, the confusion of *Anopheles* spp. with *Aedes* spp. mosquitoes as the primary vector of DENV; or knowledge of the peak biting times of the *Aedes* spp. mosquitoes (S1 Information). As a result, these physicians may disseminate the incorrect information, creating confusion amongst the community due to different messages provided by health workers in the preventive medicine program, thereby reducing the overall benefit of community-based health education on dengue control [33].

In Vietnam, the national health system requires that all physicians, both general and specialized (in any field), must work at outpatient clinics on rotation. Physicians may also provide medical consultation at their own private office after their normal working time. As a result, dengue patients presenting to outpatient clinics may be seen not only by infectious disease specialists or pediatricians, but by those specializing in internal medicine, emergency medicine or general practitioners, for example. Such physician demographics were represented in our survey, with 50% of physicians being specialized in fields other than infectious diseases and pediatrics. Accordingly, without a background in infectious diseases or pediatric illnesses, the main focus of the physician is more likely to be on patients’ immediate well-being as opposed to dissemination of public health messages that aim to reduce subsequent transmission. Moreover, due to the very small number of physicians available to treat a very large number of people within the general public (only 7.2 physicians (in all fields) per 10,000 population in 2010 [34]), the high case load of suspected dengue patients presenting at the outpatient clinics every day, and the limited time available to spend with each patient, it is often challenging for physicians to dispense clinical advice, sometimes combined with public health messages about DENV transmission. Nonetheless, as per the WHO guidelines in dengue diagnosis and treatment [35], each suspected dengue patient, who presents at the outpatient clinics during the early phase of their illness, should come back for follow-up visits during their illness. Therefore, these recurrent visits provide additional opportunity for physicians not only to discuss with patients and caregivers about patients’ illness, but also about how to prevent further DENV transmission. To assist physicians and other health workers (nurses, assistant physicians) in meeting these demands, health care services can develop more effective strategies to communicate practical dengue control measures, and make these available to health workers in outpatient clinics, for distribution to members of dengue-affected households. Leaflets and booklets are useful for communicating clear and succinct information to patients about dengue and minimizing risk of DENV transmission. Further, this information can be kept on hand, and used as a reference, encouraging preventive measures to reduce further DENV transmission.

Interestingly however, despite some patients having incomplete knowledge about the specifics of DENV transmission, the majority of patients and their families had an acute awareness of DENV and knowledge of ways to prevent further transmission in the household. For instance, misunderstanding about the peak biting times of *Ae*. *aegypti* probably explains the reliance on mosquito bed nets by some families, a practice unlikely to significantly reduce the risk of being bitten by *Ae*. *aegypti* mosquitoes, and therefore the risk of DENV infection, since these mosquitoes are day-time feeders, with peak biting periods early in the morning and evening before dusk [1]. Yet, most patients reported avoiding mosquito bites and killing adult mosquitoes to avoid further DENV transmission.

Despite the finding that majority of physicians reported a sense of responsibility for educating patients about DENV transmission, more than half of the patients said they did not receive, could not recall, or apparently misunderstood some specific information from their physicians during the consultations. Both time constraints, and sometimes a lack of specific knowledge on DENV transmission among physicians can prevent patients being provided with the required knowledge to maximize effective vector control in their homes, and hence minimize focal DENV transmission within households [36]. Support from the health system through provision and distribution of brochures and leaflets at outpatient clinics may alleviate this problem, by relaying consistent and accurate advice.

The disparity in the responses of physicians and families about DENV transmission and prevention is likely due to several factors. First, the physicians were likely to respond to questions on their preventive practice based on what they consider to be ideal responses, as opposed to what they actually do in their daily clinical practice, so these results need to be interpreted conservatively. Second, these results suggest that patients and families may have failed to understand or recall all the information presented by their physicians during the consultation. On the other hand, information on DENV transmission and its prevention are also periodically disseminated to community, especially before and during dengue season, by auxiliary health workers at the commune level and preventive medicine centers using posters, brochures, leaflets or multimedia tools (e.g. radio, television). Therefore, even though patients and their families did not receive any recommendation from their physicians during the medical consultation, they may voluntarily act to reduce risk of DENV infections in community. Methods for assessing the behavioral impact of these communication tools, such as the Communication for Behavioral Impact (COMBI) methodology [37], should be employed to measure the effectiveness of communication tools that promote vector control behavior in the community.

Participants in the patient group KAP survey were recruited from among subjects already enrolled into other dengue research studies being conducted by our group; thus dedicated study physicians (who did not participate in the physician survey) had provided information about dengue to these participants or their family members during the informed consent process less than 2 weeks prior to presentation of our survey. Therefore, the information that patients received from these study physicians was likely to have been more detailed than that received by the average patient at a local clinic. Despite this, results indicate that the understanding and/or recollection of information disseminated by their physicians during the consultation by patients was incomplete. Further, although the patient responses to the KAP survey here may not be representative of the general experience of most dengue patients when seeking treatment, there is no reason to suggest that their motivation to act upon their physician’s recommendations are any different to the general public. It is important to note that none of the physicians participating in our survey were involved in either of the two dengue studies through which we recruited participants into the patient group. A further study with a larger sample size is needed to better understand the actual situation regarding KAP in different health care levels, on dissemination of medical and practical information relating to DENV infection and transmission.

In the KAP survey, the use of insect repellent was one of the common measures that physicians recommended to their patients to help protect both patients and other relatives in the household from mosquito bites. Insect repellents have been demonstrated to effectively repel insects, among which, DEET-based preparations provide the greatest protection [23–29]. In testing the efficacy of a locally available 13% DEET-based repellent, however, we found no difference in the duration of protection from the DEET-based repellent between dengue patients and healthy control participants. This suggests that DENV infection, and the accompanying fever, does not reduce the repellent’s effectiveness, and personal repellent use by both the dengue patient, and people living in the same household, should continue to be a public health recommendation. This is the first study in which the effectiveness of mosquito repellent on febrile dengue patients, as compared to healthy controls, has been tested. Although dengue patients in this study were in days 3–6 of illness, we know that dengue patients can be infectious to mosquitoes even in the first days of illness, and before they become symptomatic [8,11]. We therefore advocate an integrated approach to stop focal transmission in the home; *frequent* use of a topical repellent by the patient will stop new mosquito infections, while killing existing mosquitoes (that were potentially already exposed to the virus) can prevent subsequent infections in naïve human hosts.

The duration of effectiveness of our chosen DEET-based mosquito repellent on non-febrile healthy participants was shorter than that of other products reported in other studies, as well as that stated by manufacturer on the product label (up to 10 hours protection) [23–29]. Differences in formulation and strength are known to alter the protective duration of repellent activity [38–40]. Variations in efficacy of mosquito repellent may be influenced by a variety of biological and non-biological factors, such as environmental conditions (light, temperature, humidity) [41], experimental procedures (repellent dose, exposure time, mosquito cage size) [42,43], mosquito age, species and strain [44,45]; as well as the attractiveness of individual volunteers [30,31,41,45,46]. Despite this, even with a higher concentration of DEET (13% versus 4.75%), and a larger volume of repellent (2 mL versus 1 mL), the product we used protected participants from a host-seeking mosquito for a relatively shorter duration (00h49m32s versus 01h28m24s) than the commercial DEET-based repellent (OFF! Skintastic for Kids), reported in the study of Fradin *et al*. [26]. The product we tested also provided a considerably shorter duration of protection (00h49m32s versus 04h00m00s) than a more comparable 15% DEET-based product (OFF! Active), used by Bissinger *et al*. [23]. Therefore, while our study advocates that physicians’ continue to recommend the use of insect repellent to protect dengue patients as well as other family members from mosquito bites, our data suggest that the frequency of application should be considerably greater than that stipulated on the label to maintain effective protection.

In conclusion, our KAP survey highlights the importance of physicians, as well as other heath care workers, being adequately knowledgeable about dengue and the DENV transmission cycle, because these data indicate that patients respect, and adhere to the practical advice provided by their consulting physician. In addition to knowledge gaps among physicians, we have demonstrated that insufficient consultation time to disseminate information is a common and important problem. These issues become barriers to what could otherwise be an effective medium for spreading practical information about preventing DENV transmission to those who would benefit from it most. The results of this survey can be used to help develop a suitable training programme for physicians involved in dengue case management. Patients and caregivers also failed to recall information from their physicians, likely a result of brief physician consults and/or ineffective modes of communication. In a region like Southern Vietnam where DENV is *hyperendemic* year-round, a combination of targeted messaging, and community-wide education campaigns would benefit all community residents. Future surveys should compare effectiveness of communication methods to enhance the quality and frequency of interventions that householders can apply to prevent further dengue transmission in their home and community.

The use of insect repellent was commonly recommended by physicians to protect dengue patients and their family members from mosquito bites. Our repellent experiments therefore investigated DEET-based mosquito repellent, and found that it provides similar duration of protection to febrile and afebrile people against mosquito bites. We advocate that physicians continue to recommend the application of DEET-based repellent to avoid mosquito bites, however that this application be *more frequent* than that on the manufacturer’s label. This, in conjunction with active vector control efforts in the home should help to subsequently reduce the risk of further DENV transmission. Moreover, findings from the KAP survey and mosquito repellent experiments can also be used to assist developing effectively vector control for DENV and other flaviviruses transmitted by *Aedes* spp.

## Supporting Information

S1 Fig Schedule and duration of exposures to *Aedes aegypti* mosquitoes. Each exposure to a cage of ten mosquitoes lasted for 2 minutes. If a mosquito failed to land on participants’ skin (for at least 2 seconds) within the 2 minutes of each exposure, subsequent exposures occurred at staggered intervals thereafter. The order of mosquito cages used on the repellent-treated arm was determined randomly. If the mosquitoes failed to land on the repellent-treated arm after a maximum of 245 minutes (4 hours and 5 minutes), the experiment was finished, with the data point for failed landing being ‘censored’ at the 245-minute mark.
(TIF)

S1 Information Questionnaire for physicians. The correct answers to the questions in the knowledge theme are highlighted in bold. At the right of each response is the number of participants that selected that response, and the associated percentage.
(PDF)

S2 Information Questionnaire for patients. The correct answers to the questions in the knowledge theme are highlighted in bold. At the right of each response is the number of participants that selected that response, and the associated percentage.
(PDF)
