# Supplementary figures and images for: Physicians, Primary Caregivers and Topical Repellent: All Under-Utilised Resources in Stopping Dengue Virus Transmission in Affected Households
Source: PLoS Negl Trop Dis. 2016 May 10;10(5):e0004667. doi: 10.1371/journal.pntd.0004667 (PMC4862674; doi:10.1371/journal.pntd.0004667)

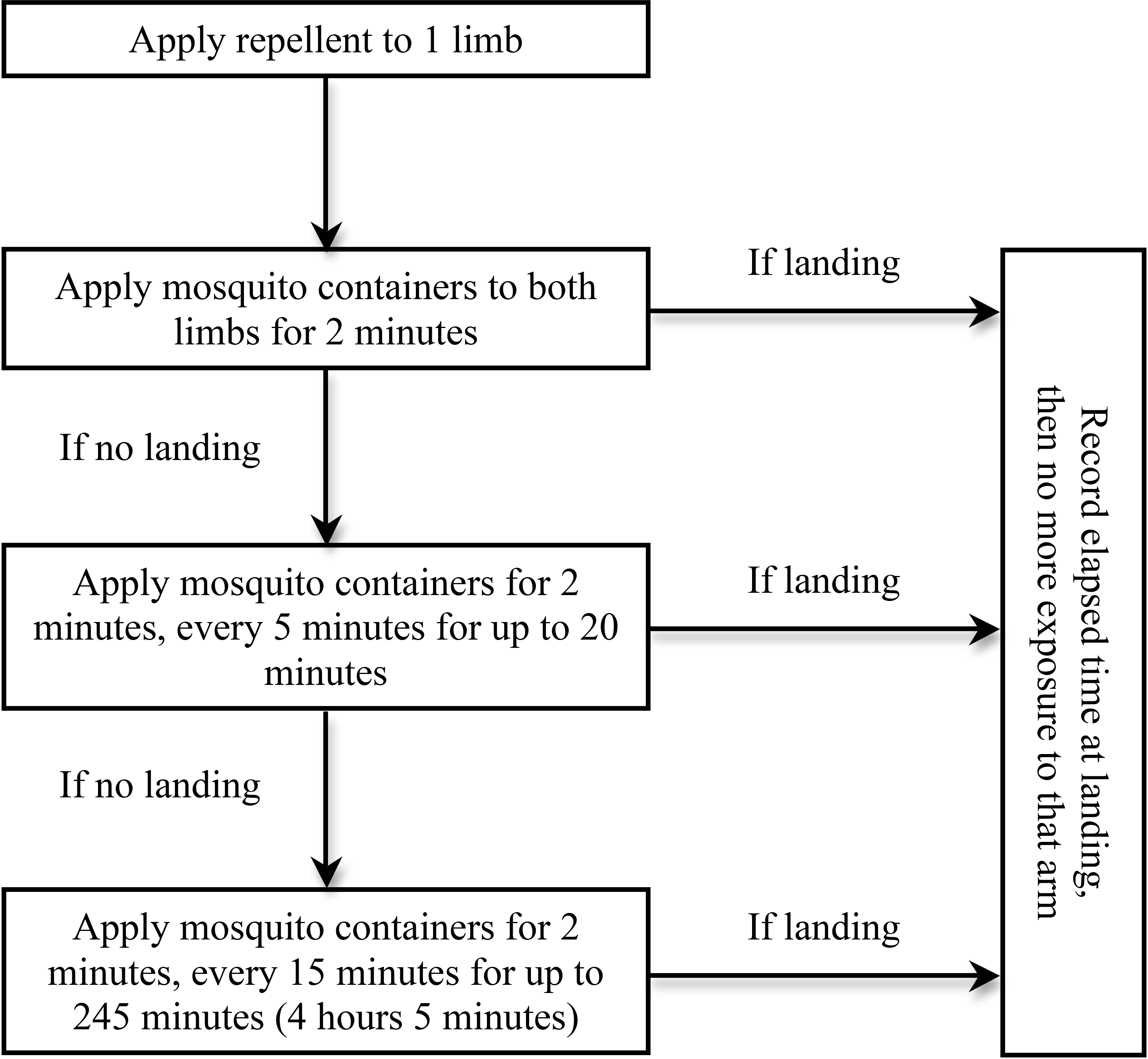

Supplement: S1 Fig — Each exposure to a cage of ten mosquitoes lasted for 2 minutes. If a mosquito failed to land on participants’ skin (for at least 2 seconds) within the 2 minutes of each exposure, subsequent exposures occurred at staggered intervals thereafter. The order of mosquito cages used on the repellent-treated arm was determined randomly. If the mosquitoes failed to land on the repellent-treated arm after a maximum of 245 minutes (4 hours and 5 minutes), the experiment was finished, with the data point for failed landing being ‘censored’ at the 245-minute mark. (TIF) [file pntd.0004667.s001.tif]
