# Supplementary material for: Physicians, Primary Caregivers and Topical Repellent: All Under-Utilised Resources in Stopping Dengue Virus Transmission in Affected Households
Source: PLoS Negl Trop Dis. 2016 May 10;10(5):e0004667. doi: 10.1371/journal.pntd.0004667 (PMC4862674; doi:10.1371/journal.pntd.0004667)
Supplement: S1 Information — The correct answers to the questions in the knowledge theme are highlighted in bold. At the right of each response is the number of participants that selected that response, and the associated percentage. (PDF) [file pntd.0004667.s002.pdf]

QUESTIONNAIRE – PHYSICIANS

Knowledge, Attitude and Practice on dengue virus transmission and prevention:

1. *Dengue is a very important disease in your day-to-day clinical work?*
   a. [__] Agree (46/50 – 92%)
   b. [__] Disagree (4/50 – 8%)

2. *Only some serotypes of dengue virus can cause disease in the Vietnam.*
   a. [__] Agree (26/50 – 52%)
   b. [__] **Disagree (20/50 – 40%)**
   c. [__] Don't know (4/50 – 8%)

3. *For how long do you think dengue patients can be infectious to mosquitoes?*
   a. [__] 1-2 days (4/50 – 8%)
   b. [__] 1-2 weeks (19/50 – 38%)
   c. [__] For the duration of the febrile period (21/50 – 42%)
   d. [__] Don't know (6/50 – 12%)

4. *Which of the following best describes the vector of dengue viruses?*
   a. [__] Anopheles sp. mosquitoes primarily transmit dengue viruses (7/50 – 14%)
   b. [__] The mosquitoes that transmit dengue viruses rest mainly outdoors (0)
   c. [__] **The mosquitoes that transmit dengue viruses breed mainly in artificial water containers, e.g. water jars or discarded containers (34/50 – 68%)**
   d. [__] The mosquitoes that transmit dengue viruses bite during the day and night (9/50 – 18%)

5. *When mosquitoes feed on a dengue case, which of the following is true?*
   a. [__] **No infection of the mosquito if the dengue virus concentration in the blood is too low** (14/50 – 28%)
   b. [__] The mosquito becomes immediately infected and can infect others when it next feeds (29/50 – 58%)
   c. [__] The mosquito will live for another 2-3 months (2/50 – 4%)
   d. [__] The female mosquito will spread the dengue virus to other mosquitoes when they mate (5/50 – 10%)

6. *There is an effective and available vaccine for dengue and Vietnamese children should be immunized when they are young.*
   a. [__] Agree (4/50 – 8%)
   b. [__] **Disagree (41/50 – 82%)**
   c. [__] Don't know (5/50 – 10%)

You meet a patient with clinically suspected dengue at your clinic,

7. *Do you think that discussing dengue virus transmission and prevention with the family is your responsibility?*
   a. [__] Agree (47/50 – 94%)
   b. [__] Disagree (2/50 – 4%)
   c. [__] Don't know (1/50 – 2%)

8. *Do you usually (i.e. most times) discuss with the family how the patient was infected, i.e. bitten by a dengue virus infected mosquito?*
   a. [__] Yes (48/50 – 96%)
   b. [__] No (2/50 – 4%)

9. *If the patient is being managed on an ambulatory basis, do you usually (i.e. most times) suggest actions that the patient/family should take to stop the patient being bitten by mosquitoes when they are at home?*
   a. [__] Yes (41/50 – 82%)
   b. [__] No ***(if No, skip to Q. 11) (9/50 – 18%)***
   c. [__] Don't know

10. *If YES, what actions do you usually (i.e. most times) recommend? (choose* ***ALL*** *that apply)*
   a. [__] Mosquito net (41/41 – 100%)
   b. [__] Mosquito bat killer (28/41 – 68%)
   c. [__] Insecticidal spraying (32/41 – 78%)
   d. [__] Mosquito repellent (32/41 – 78%)
   e. [__] Other, please specify: *remove the discarded water containers (bottles, vases, etc.) and cut down bushes* (7); *wear long sleeve shirt and pants* (1); *cut down bushes and keep the living environment clean* (1); *long sleeve shirt and pants, remove breeding sites of mosquitoes* (1); *people should not be outdoor as dusk is falling* (1)

11. *Do you usually (i.e. most times) give advice on what actions could be taken to try to prevent other people living with the patient from getting dengue?*
   a. [__] Yes (44/50 – 88%)
   b. [__] No ***(if No, skip to Q. 15) (6/50 – 12%)***
   c. [__] Don't know (0)

12. *If YES, what actions do you recommend? (choose* ***ALL*** *that apply)*
   a. [__] Kill mosquitoes (36/44 – 81.8%)
   b. [__] Remove breeding sites of mosquitoes and larvae (43/44 – 97.7%)
   c. [__] Avoid mosquito bites (38/44 – 86.4%)
   d. [__] Other, please specify: *mosquito net* (2); *mosquito net and insecticidal spraying* (1)

13. *How much time do you usually spend to discuss with your patient and her/his family about her/his illness?*
   a. [__] Less than 1 minute (4/50 – 8%)
   b. [__] 1 – 3 minutes (28/50 – 56%)
   c. [__] More than 3 minutes (18/50 – 36%)

14. *By which method do you usually give advice to your patients on dengue virus transmission and prevention? (choose **ALL** that apply)*
   a. [__] Verbal (50/50 – 100%)
   b. [__] Leaflet (22/50 – 44%)
   c. [__] Booklet (2/50 – 4%)

15. *What barriers do you meet when discussing how dengue is transmitted with patients? (choose **ALL** that apply)*
   a. [__] Not enough time (44/50 – 88%)
   b. [__] Patient or their family is not interested (18/50 – 36%)
   c. [__] Not confident that I will give the correct information (6/50 – 12%)
   d. [__] Others, please specify: *forget the role of transferring information on dengue virus transmission and prevention* (1); *no training or guidelines on transferring information on dengue virus transmission and prevention* (1)

Demographic information of the physician

16. *Age*: [__|__] years

17. *Sex*: [__] Male [__] Female

18. *Which of the following best describes your area of specialty?*
   a. [__] Pediatric medicine
   b. [__] Infectious diseases
   c. [__] Others, please specify: ___________________________

19. *How often do you work at the outpatient clinic*
   a. [__] Every day
   b. [__] One day per week
   c. [__] Two days per week
   d. [__] Three days per week
   e. [__] Four days per week
   f. [__] Never
   g. [__] Other, please specify: __________________

20. *What sector do you usually work in?*
   a. [__] Public sector
   b. [__] Private sector
   c. [__] Both

21. *Do you work in your own private office?*
   a. [__] Yes
   b. [__] No
