# Supplementary material for: Physicians, Primary Caregivers and Topical Repellent: All Under-Utilised Resources in Stopping Dengue Virus Transmission in Affected Households
Source: PLoS Negl Trop Dis. 2016 May 10;10(5):e0004667. doi: 10.1371/journal.pntd.0004667 (PMC4862674; doi:10.1371/journal.pntd.0004667)
Supplement: S2 Information — The correct answers to the questions in the knowledge theme are highlighted in bold. At the right of each response is the number of participants that selected that response, and the associated percentage. (PDF) [file pntd.0004667.s003.pdf]

QUESTIONNAIRE – PATIENTS

Knowledge, Attitude and Practice on dengue virus transmission and prevention:

You recently visited a physician at CH#1, CH#2 or HTD because you/your child had fever. In the next 3 MINUTES, I would like to ask you some general questions about dengue.

1. *Dengue is a common disease in Vietnam:*
   a. [__] **Agree (40/49 – 82%)**
   b. [__] Disagree (1/49 – 2%)
   c. [__] Don't know (8 – 16%)

2. *Dengue is more common in children than adults:*
   a. [__] **Agree (33/49 – 67.35%)**
   b. [__] Disagree (5/49 – 10.2%)
   c. [__] Don't know (11/49 – 22.45%)

3. *Dengue can be a serious, even fatal disease:*
   a. [__] **Agree (47/49 – 95.9%)**
   b. [__] Disagree (0)
   c. [__] Don't know (2/49 – 4.1%)

4. *Dengue can spread between humans by sneezing or coughing:*
   a. [__] Agree (1/49 – 2%)
   b. [__] **Disagree (30/49 – 61%)**
   c. [__] Don't know (18/49 – 37%)

5. *Dengue can be spread between humans by mosquitoes:*
   a. [__] **Agree *(go to question 6) (47/49 – 95.9%)***
   b. [__] Disagree ***(go to question 12) (0)***
   c. [__] Don't know ***(go to question 12) (2/49 – 4.1%)***

6. *All types of mosquitoes can spread dengue:*
   a. [__] Agree (2/47 – 4.3%)
   b. [__] **Disagree (41/47 – 87.2%)**
   c. [__] Don't know (4/47 – 8.5%)

7. *The mosquitoes that spread dengue bite humans mainly at night:*
   a. [__] Agree (28/47 – 59.6%)
   b. [__] **Disagree (10/47 – 21.3%)**
   c. [__] Don't know (9/47 – 19.1%)

8. *The mosquitoes that spread dengue breed in containers carrying fresh water:*
   a. [__] **Agree (34/47 – 72.3%)**
   b. [__] Disagree (7/47 – 14.9%)
   c. [__] Don't know (6/47 – 12.8%)

9. *Do you routinely take action to kill mosquitoes or reduce breeding places for mosquitoes in or around your house?*
   a. [__] Yes ***(go to question 10) (36/47 – 76.6%)***
   b. [__] No ***(go to question 12) (11/47 – 23.4%)***
   c. [__] Don't know ***(go to question 12) (0)***

10. *If YES, how often do you take these actions:*
   a. [__] Every day (24/36 – 66.7%)
   b. [__] At least once per week (12/36 – 33.3%)
   c. [__] At least once per month (0)
   d. [__] At least once per year (0)
   e. [__] Don't know (0)

11. *What do you focus on?*
   a. [__] Killing adult mosquitoes (9/36 – 25%)
   b. [__] Removing breeding sites and larvae ("wrigglers") (10/36 – 27.8%)
   c. [__] Both (17/36 – 47.2%)
   d. [__] Don't know (0)

The physician at CH#1, CH#2 or HTD thought it was possible that you/your child had dengue. In the next 3 MINUTES, I would like to ask you some specific questions about dengue and your visit to the physician.

12. *Did the physician you visited at CH#1, CH#2 or HTD mention dengue as a possible diagnosis for you/your child?*
   a. [__] Yes (30/49 – 61.2%)
   b. [__] No (19/49 – 38.8%)
   c. [__] Don't know (0)

13. *Did the physician give you information on your/your child's illness in a way that you could easily understand?*
   a. [__] Yes (42/49 – 85.7%)
   b. [__] No (7/49 – 14.3%)
   c. [__] Don't know (0)

14. *Did your physician discuss with you how dengue is spread between humans?*
   a. [__] Yes (18/49 – 36.7%)
   b. [__] No (31/49 – 63.3%)
   c. [__] Don't know (0)

15. *Did your physician suggest that others in your household were at risk of getting dengue?*
   a. [__] Yes (20/49 – 40.8%)
   b. [__] No (29/49 – 59.2%)
   c. [__] Don't know (0)

16. *Did your physician suggest actions that you or others in your household (e.g. other children) should take to limit their risk of getting dengue?*
    a. [__] Yes ***(go to question 17) (15/49 – 30.6%)***
    b. [__] No ***(go to question 21) (34/49 – 69.4%)***
    c. [__] Don't know ***(go to question 21) (0)***

17. *If YES, what actions were recommended? (choose **ALL** that apply)*
    a. Killing mosquitoes (12/15 – 80%)
    b. Remove the breeding places of mosquitoes and larvae ("wrigglers") (8/15 – 53.5%)
    c. Avoiding mosquito bites (14/15 – 93.3%)
    d. Other, please specify:……………………………………………………… (0)

18. *Did you or others in your house perform the actions suggested by your physician in the 3 days after you/your child visited the physician?*
    a. [__] Yes ***(go to question 20) (15/15 – 100%)***
    b. [__] No ***(go to question 19) (0)***
    c. [__] Don't know ***(go to question 19) (0)***

19. *If NO, did you or others in your house perform any other actions that you thought could stop dengue being transmitted in or around your home in the 3 days after you/your child visited the physician?*
    a. [__] Yes ***(go to question 20) (0 – skipped)***
    b. [__] No ***(go to question 21) (0 – skipped)***
    c. [__] Don't know ***(go to question 21) (0 – skipped)***

20. *If YES, what actions were taken? (choose **ALL** that apply)*
    a. Killing mosquitoes (9/15 – 60%)
    b. Remove the breeding places of mosquitoes and larvae ("wrigglers") (6/15 – 40%)
    c. Avoiding mosquito bites (13/15 – 86.7%)
    d. Other, please specify: *isolate the dengue patient from the other family members* (1/15 – 6.7%)

21. *Did your physician suggest specific actions to stop you/your sick child from being bitten by mosquitoes?*
    a. [__] Yes (10/49 – 20.4%)
    b. [__] No (39/49 – 79.6%)
    c. [__] Don't know (0)

22. *Did you or others in your house perform any actions that you thought could stop you/your sick child from being bitten by mosquitoes?*
    a. [__] Yes (42/49 – 85.7%)
    b. [__] No (7/49 – 14.3%)
    c. [__] Don't know (0)

23. *If YES, what actions were taken? (choose **ALL** that apply)*
    a. [__] Mosquito net (35/42 – 83.3%)
    b. [__] Mosquito bat killers (12/42 – 28.6%)
    c. [__] Insecticidal spraying (24/42 – 57.1%)
    d. [__] Mosquito repellent (7/42 – 16.7%)
    e. [__] Other: *mosquito repelling coil* (3); *remove breeding sites of mosquitoes* (6); *remove breeding sites of mosquitoes and use coil* (1)

Demographic information:

24. *Age:* [__|__] years

25. *Sex:* [__] Male [__] Female

26. *Are you the person who has dengue?*
    a. [__] Yes
    b. [__] No

27. *If No, Who is it? ………………………………………*

28. *What is your job?*
    a. Vendor
    b. Worker
    c. Housework woman/man
    d. Office staff
    e. Teacher
    f. Farmer
    g. Other, please specify: ………………..

29. *Which of the following best describes your education level?*
    a. Illiterate
    b. Elementary school
    c. High school
    d. College or higher
